# Genetic Diversity of SARS-CoV-2 over a One-Year Period of the COVID-19 Pandemic: A Global Perspective

**DOI:** 10.3390/biomedicines9040412

**Published:** 2021-04-11

**Authors:** Miao Miao, Erik De Clercq, Guangdi Li

**Affiliations:** 1 Hunan Provincial Key Laboratory of Clinical Epidemiology, Xiangya School of Public Health, Central South University, Changsha 410078, China; miaomiao19@csu.edu.cn; 2 Rega Institute for Medical Research, Department of Microbiology, Immunology and Transplantation, KU Leuven, Herestraat 49, B-3000 Leuven, Belgium; erik.declercq@kuleuven.be

**Keywords:** SARS-CoV-2, COVID-19, global pandemic, genetic diversity, genetic variant

## Abstract

Severe acute respiratory syndrome coronavirus 2 (SARS-CoV-2) caused a global pandemic of coronavirus disease in 2019 (COVID-19). Genome surveillance is a key method to track the spread of SARS-CoV-2 variants. Genetic diversity and evolution of SARS-CoV-2 were analyzed based on 260,673 whole-genome sequences, which were sampled from 62 countries between 24 December 2019 and 12 January 2021. We found that amino acid (AA) substitutions were observed in all SARS-CoV-2 proteins, and the top six proteins with the highest substitution rates were ORF10, nucleocapsid, ORF3a, spike glycoprotein, RNA-dependent RNA polymerase, and ORF8. Among 25,629 amino acid substitutions at 8484 polymorphic sites across the coding region of the SARS-CoV-2 genome, the D614G (93.88%) variant in spike and the P323L (93.74%) variant in RNA-dependent RNA polymerase were the dominant variants on six continents. As of January 2021, the genomic sequences of SARS-CoV-2 could be divided into at least 12 different clades. Distributions of SARS-CoV-2 clades were featured with temporal and geographical dynamics on six continents. Overall, this large-scale analysis provides a detailed mapping of SARS-CoV-2 variants in different geographic areas at different time points, highlighting the importance of evaluating highly prevalent variants in the development of SARS-CoV-2 antiviral drugs and vaccines.

## 1. Introduction

**Figure 1:**
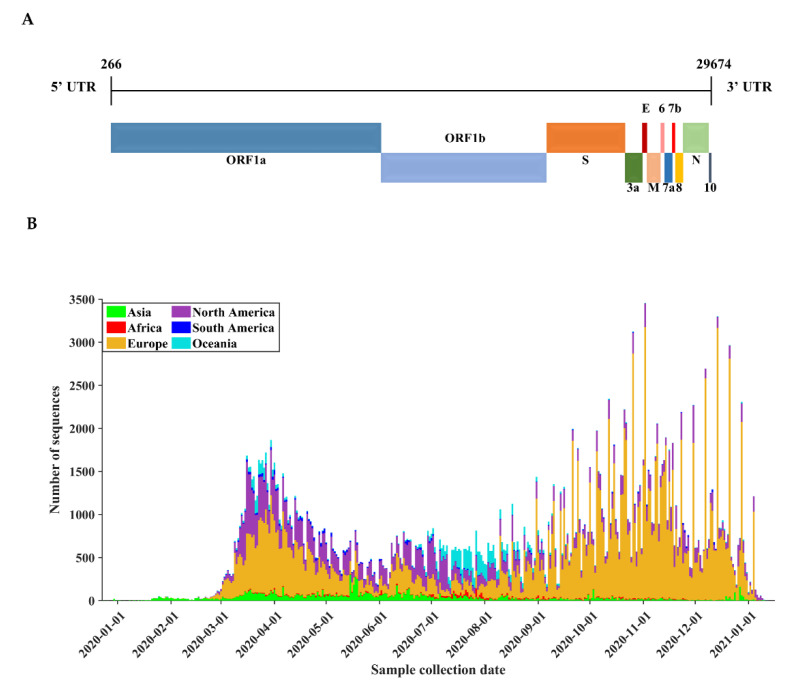
Severe acute respiratory syndrome coronavirus 2 (SARS-CoV-2) genome organization and the distribution of the SARS-CoV-2 genome sequence used in this study. (**A**) Genomic architecture of SARS-CoV-2 based on the reference sequence (Wuhan-Hu-1, NCBI accession NC_045512). (**B**) The temporal and geographic distribution of all sequences. All temporal analyses were based on the date of sequence collection.

Since December 2019, severe acute respiratory syndrome coronavirus 2 (SARS-CoV-2) has caused a global pandemic. As of 16 January 2021, more than 93 million cases and 2 million deaths have been reported, according to the daily update of the World Health Organization. As a virus of the *Coronaviridae* family, SARS-CoV-2 is a positive-sense, single-stranded RNA virus whose genome contains approximately 30,000 nucleotides, making it one of the largest genomes among RNA viruses. The coding region of the SARS-CoV-2 genome encodes for nonstructural proteins (NSPs) and structural proteins in different open reading frames (ORFs). The genetic makeup of polyproteins pp1a and pp1ab, encoded by ORFs 1a and 1b at the 5′-end, contains 16 nonstructural proteins (numbered from NSP1 to NSP16). The 3′-end of the genome (21 to 29 kb) encodes for at least 6 accessory proteins (ORF3a, ORF6, ORF7a, ORF7b, ORF8, ORF10) [1] and 4 structural proteins: spike glycoprotein (S), envelope protein (E), membrane glycoprotein (M), and nucleocapsid protein (N) (Figure 1A). Since viral proteins such as RNA-dependent RNA polymerase and spike glycoprotein are key drug targets [2], genetic variations in the genome may exert an important impact on the efficacy of current vaccines and antiviral treatments [3].

Global surveillance of SARS-CoV-2 genetic variants is important to control the coronavirus disease 2019 (COVID-19) pandemic. Previous analyses of SARS-CoV-2 genomes have characterized proximal origin [4,5,6], evolutionary rates [7], spatiotemporal distributions [8], transmission dynamics in many countries [9,10,11,12,13,14,15], phylogenetic networks of dominant strains [16,17,18,19], intragenomic diversity [20], epistatic interactions between viral genes [21], genetic variants in response to disease severity and immune responses [22,23,24,25], and the impact of spike variants on current vaccines and antibodies [26,27,28,29,30]. Despite its long genome structure, SARS-CoV-2 encodes a 3′-to-5′ exoribonuclease called NSP14 that provides the proofreading activity to correct errors made by the viral RNA-dependent RNA polymerase, thereby promoting a high fidelity of genome replication [31,32]. Nevertheless, some variants, such as D614G in spike, can escape proofreading and significantly shape the global dispersal of SARS-CoV-2 infections [33]. Moreover, circulating variants may exert a potential impact on the development of vaccines and antiviral drugs to control the COVID-19 pandemic [34]. Due to the dynamic dispersal of viral strains worldwide, it remains important to continue the surveillance of SARS-CoV-2 variants from a global perspective [35].

Here, we have conducted global surveillance of circulating variants in SARS-CoV-2 based on a large-scale dataset of whole-genome sequences from 62 countries worldwide. For the development of novel vaccines and antiviral drugs, this study contributes to a deeper understanding of diversity and global surveillance of key viral variants of SARS-CoV-2.

## 2. Materials and Methods

### 2.1. Data Collection

On 16 January 2021, we extracted SARS-CoV-2 complete genome sequences with high coverage (with <1% undefined bases and <0.05% unique amino acid (AA) substitutions and no insertion/deletion unless verified by submitter) from the GISAID database (https://www.gisaid.org/, the accessed date: 16 January 2021). We also downloaded the sequence metadata regarding the geographical locations and collection times. We acknowledge all contributors who have kindly deposited and shared their genome data on GISAID. A list of genome sequence acknowledgments is provided in Data S1. The complete genome of the Wuhan-Hu-1 isolate (NCBI accession NC_045512) was also collected as a reference strain.

Sequence quality control was conducted as follows: (i) one sequence per patient was sampled, while multiple sequences sampled from one patient were screened using the metadata regarding patient ID, authors, lab origin, and country origin; and (ii) sequences from nonhuman origins were removed.

### 2.2. Multiple Sequence Alignment

Nucleotide sequences were aligned to the reference sequence (Wuhan-Hu-1) using the option of “addfragments” in MAFFT version 7.471 [36], and output alignments were manually scrutinized by Seaview version 5.0 (http://doua.prabi.fr/software/seaview, the accessed date: 16 January 2021). The 5′ and 3′ untranslated regions were trimmed, and only sequences that contained full-length coding regions were retained. Inhouse MATLAB code (available at https://github.com/MiaoMiaorrk/sequence_analysis, the accessed date: 16 January 2021) was prepared to extract the nucleotide regions of 26 viral proteins, while the coding region of the viral polymerase NSP12 was concatenated by considering programmed −1 ribosomal frameshifting. The corresponding amino acid sequences were subsequently obtained for analyzing protein diversity and amino acid variants.

### 2.3. Protein Sequence Diversity

Polymorphic sites are amino acid or nucleotide positions that have at least one substitution compared with the reference Wuhan-Hu-1, ignoring gaps and ambiguities [30]. Given multiple sequence alignments, the amino acid substitution rate was defined as:

(1) $$Substitution\;rate=\frac{\sum_{i=1}^{L}\sum_{j=1}^{N}Setequal\left(A\left(i,j\right),A\left(i,0\right)\right)}{N\cdot L-N_{Invalid}}$$

(2) $$Setequal\left(a,b\right)=\{\begin{array}{c} 1\;,\;if\;a\neq b\;and\;a,b\;are\;both\;valid;\\ 0\;,\;otherwise.\;\;\;\;\;\;\;\;\;\;\;\;\;\;\;\;\;\;\;\;\;\;\;\;\;\;\;\;\;\;\;\;\;\;\;\;\;\;\;\;\;\;\; \end{array}$$

A(i,j) is the element at the i position of the $j_{th}$ sequence, and A(i,0) is the element at the i position of the reference Wuhan-Hu-1. L is the length of the protein, and N is the number of sequences except for the reference sequence. $N_{Invalid}$ is the total number of invalid positions containing gaps or ambiguous letters. Setequal(A(i,j),A(i,0)) is the function that checks if two elements A(i,j) and A(i,0) are equal.

### 2.4. Selective Pressure Analysis

To analyze the selective pressure on the protein-coding regions of SARS-CoV-2, the dN/dS ratio that measures the nonsynonymous (dN) to synonymous (dS) substitution rates at the coding region was calculated using KaKs_Calculator 2.0 with the method of modified YN (MYN) and the genetic code in Table 1 (standard code) [37]. The positive selection was defined by dN/dS > 1.

### 2.5. Phylogenetic Analysis

At each sample collection date, from 24 December 2019 to 12 January 2021, three genomic sequences were randomly subsampled for each continent, while the sequences were all collected if the number of sequences was less than 3. This led to a subset of SARS-CoV-2 genome sequences (*n* = 5488). Multiple sequence alignments of this subset were subsequently imported to reconstruct a maximum-likelihood phylogenetic tree using NextStrain tools (https://nextstrain.org, the accessed date: 16 January 2021).

## 3. Results

### 3.1. Basic Characteristics of SARS-CoV-2 Genome Sequences

Our study analyzed a large-scale dataset of SARS-CoV-2 genome sequences (*n* = 260,673) that fulfilled the quality control criteria. During the pandemic of COVID-19, from 24 December 2019 to 12 January 2021, these full-length sequences were sampled from 62 countries located in Europe (*n* = 166,443), North America (*n* = 61,232), Asia (*n* = 13,709), Oceania (*n* = 13,166), South America (*n* = 3216), and Africa (*n* = 2907). The collection time of SARS-CoV-2 genome sequences is visualized in Figure 1B. Most sequences were collected during the first wave (March to May 2020) before the summer in the Northern Hemisphere and during the second wave (September 2020 to January 2021) in Europe and North America, where the COVID-19 pandemic remains severe.

### 3.2. Amino Acid Diversity of SARS-CoV-2 Proteins

The SARS-CoV-2 genome encodes 26 functional proteins that are potentially evolving during the global pandemic. Given 260,673 sequences, we measured the average amino acid diversity of SARS-CoV-2 proteins (Equation (1), Table 1). Among 26 viral proteins, the highest amino acid diversity was observed in ORF10 (0.72%), followed by nucleocapsid (0.31%), ORF3a (0.20%), spike (0.14%), NSP12 (0.13%), and ORF8 (0.12%), while the other proteins had low protein diversity (<0.1%). In contrast, two nonstructural proteins (NSP10 and NSP11) and two structural proteins (envelope and membrane proteins) had a low protein diversity (<0.02%).

**Figure 2:**
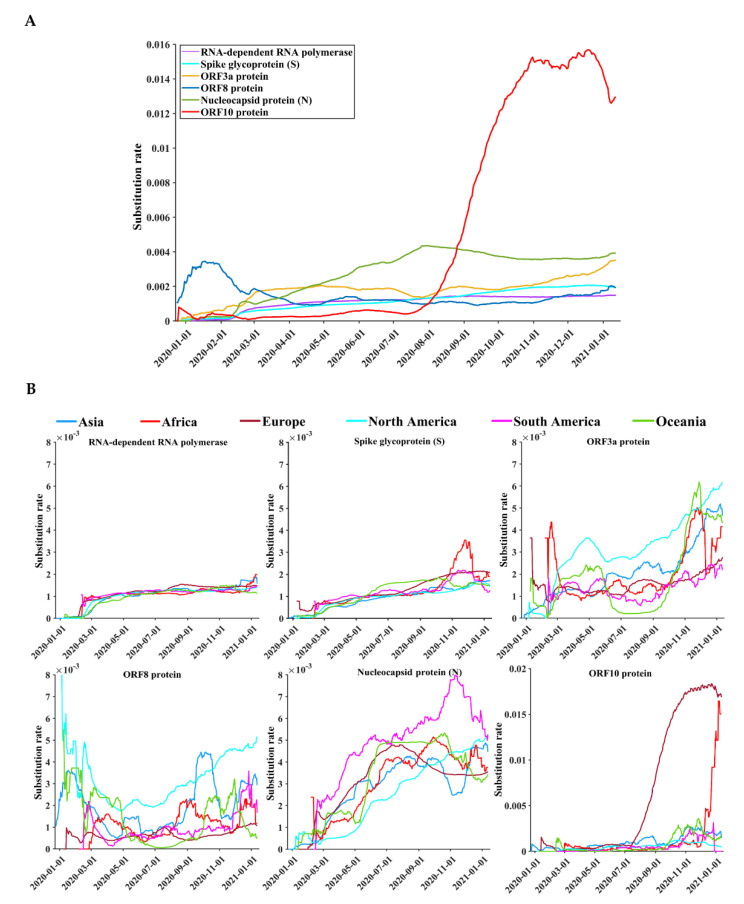
Temporal distributions of the substitution rates of the six SARS-CoV-2 proteins. The proteins were the top six proteins with the highest substitution rates. (**A**) The global substitution rate curves of SARS-CoV-2 proteins based on a time sliding window. (**B**) The substitution rate curves of SARS-CoV-2 proteins based on a time sliding window on different continents. The vertical axis represents the moving-window substitution rate, calculated by dividing the total number of polymorphic sites of a protein contained in the sequences, 15 days before and after a specific date, by the total number of all positions of the protein in the period.

Due to the worldwide spread of SARS-CoV-2 infections, dynamic patterns of protein diversity were observed over time, especially in six viral proteins: ORF10, nucleocapsid, ORF3a, spike, NSP12, and ORF8 (Figure 2A). Unlike other proteins, whose protein diversities increase gradually over time, the protein diversity of ORF10 has increased sharply since July 2020. Figure 2B shows that the highest substitution rate of the ORF10 protein appeared in Europe, followed by Africa. Although the increasing patterns of protein diversity of the remaining four proteins, from December 2019 to January 2021, were roughly the same, the temporal patterns of spike and ORF10 diversity were different between Africa and other continents (Figure 2B). Protein diversity of spike and ORF10 reached their peaks in November and December 2020, respectively.

### 3.3. Positive Selection Is Rare in SARS-CoV-2 Protein-Coding Genes

To analyze selective pressure on SARS-CoV-2 protein-coding regions, we estimated the dN/dS ratios of nonsynonymous and synonymous substitution rates (Figure S1). The median dN/dS ratio for all protein-coding genes was 0.31 (IQR: 0.24 to 0.65). All viral protein-coding genes had a median dN/dS ratio less than 1, and the highest level of median dN/dS ratio was observed in *ORF9* (0.65, IQR: 0.49 to 0.65), followed by *ORF6* (0.56) and *ORF7b* (0.50) (Table S1).

### 3.4. Circulating Variants of SARS-CoV-2

Given the large-scale dataset of 260,673 sequences, we analyzed the characteristics of nucleotide and the amino acid variants of SARS-CoV-2. The nucleotide composition of the coding region of the reference sequence Wuhan-Hu-1 was A (29.94%), T (32.08%), C (18.37%), and G (19.61%). In the coding region of the SARS-CoV-2 genome, 22,964 of 29,409 nucleotide positions (78.08%) were polymorphic, with at least one single-nucleotide polymorphism (SNP) compared with the reference Wuhan-Hu-1. Most nucleotide substitutions (94.40%) were SNPs, while indels (5.60%) were rare. Moreover, the most common nucleotide substitutions were C to T (46.27%), G to T (13.47%), A to G (11.26%), T to C (6.66%), and others (22.34%).

We next analyzed the amino acid variants of SARS-CoV-2. There were 8484 (86.95%) polymorphic sites calculated as the number of sites having one or more substitutions compared to the reference sequence (Wuhan-Hu-1, NCBI accession NC_045512) across the 9757 amino acid positions in 26 concatenated proteins. In total, 25,629 substitutions were identified at 8484 amino acid positions. Notably, 587 of 1273 fully conserved positions were mostly located in the enzymatic proteins of NSP12 (17.05%), NSP3 (15.16%), and spike (13.90%). The frequency of variants on different continents was calculated by dividing the number of sequences carrying a given variant on a specific continent by the total number of sequences on that continent; 115 polymorphic sites occurred with a frequency greater than 0.5%. Highly polymorphic sites that occurred with a frequency greater than 1% were mostly observed in nucleocapsid (3.10%), ORF10 (2.63%), ORF8 (2.48%), ORF3a (2.18%), spike (0.71%), and NSP12 (0.64%).

**Figure 3:**
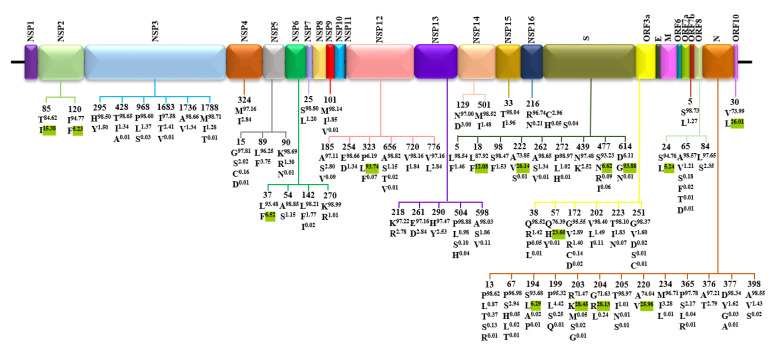
Distribution of variants at positions of SARS-CoV-2 proteins. For each site, the reference index is shown at the top, followed by variants with a frequency >1%. Variants highlighted with green superscripts were those with frequencies >5%.

In addition, the major variants of each SARS-CoV-2 protein are mapped in Figure 3. For each position, the Wuhan-Hu-1 index is shown at the top, followed by the variants with a frequency >1%. Fifteen variants with the prevalence >5% were identified, including (i) four variants in nucleocapsid: S194L (6.29%), R203K (28.45%), G204R (28.13%), and A220V (25.96%); (ii) four variants in spike: L18F (12.08%), A222V (26.14%), S477N (6.62%), and D614G (93.88%); (iii) two variants in NSP2: T85I (15.38%) and I120F (5.23%); (iv) five variants in each of the 5 proteins: L37F (6.52%) in NSP6, P323L (93.74%) in NSP12, Q57H (23.60%) in ORF3a, S24L (5.24%) in ORF8, and V30L (26.01%) in ORF10.

### 3.5. Geographical and Temporal Trends of the Frequent Variants

**Figure 4:**
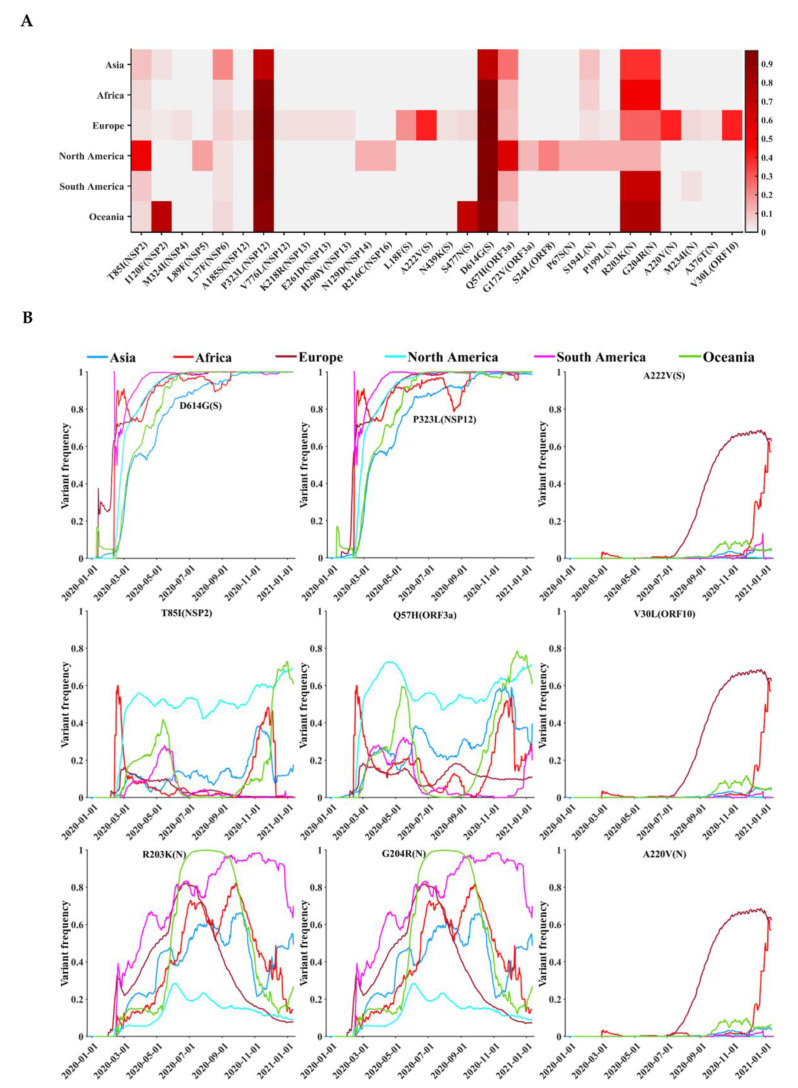
Distribution of frequent variants across the SARS-CoV-2 proteins in different geographic areas. (**A**) Frequencies of the top 30 variants, with the highest variant frequencies in different continents. (**B**) Temporal and geographic dynamics of frequent SARS-CoV-2 variants. The vertical axis represents the moving-window variant frequency calculated by dividing the number of sequences containing a specific variant, 15 days before and after a specific date, by the total number of sequences in the period (31 days).

The geographic distribution and temporal progression of frequent variants (*n* = 30), with a high frequency greater than 2.5%, are shown in Figure 4. Figure 4A shows that these variants were found mainly in nucleocapsid (P67S, S194L, P199L, R203K, G204R, A220V, M234I, and A376T), spike (L18F, A222V, N439K, S477N, and D614G), NSP12 (A185S, P323L, and V776L), NSP13 (K218R, E261D, and H290Y), NSP2 (T85I and I120F), and ORF3a (Q57H and G172V), respectively. In addition, there was one variant in each of the seven proteins NSP4 (M324I), NSP5 (L89F), NSP6 (L37F), NSP14 (N129D), NSP16 (R216C), ORF8 (S24L), and ORF10 (V30L). Among all frequent variants, D614G in spike and P323L in NSP12, with a high frequency, were common on the six continents (Figure 4A). D614G (97.26%) and P323L (97.29%) (Table 2) in South America exhibited the highest variant prevalence. R203K and G204R in nucleocapsid showed different prevalence on the six continents. Seven common variants were mainly prevalent on a single continent, including the NSP2 variant T85I in North America, the NSP2 variant I120F in Oceania, the spike variant A222V in Europe, the spike variant S477N in Oceania, the ORF3a variant Q57H in North America, the nucleocapsid variant A220V in Europe, and the ORF10 variant V30L in Europe. The prevalence of the other 19 variants was generally similar across six continents.

**Figure 5:**
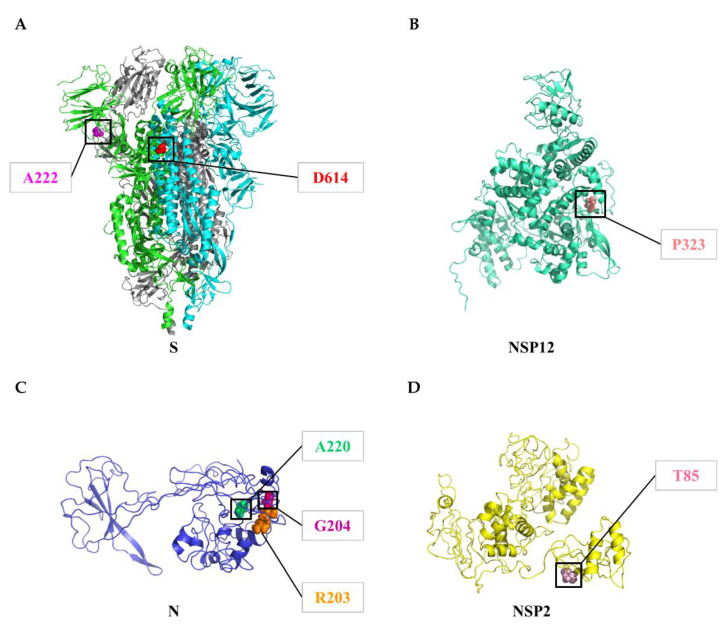
Variants in the SARS-CoV-2 proteins. (**A**) Site 222 (magenta) and Site 614 (red) of spike. Almost all sequences showed a variant (D614G). Site 614 is located at the interface between two subunits. (**B**) Site 323 (deep salmon) of the NSP12 protein. Many sequences showed a variant (P323L). (**C**) Site 203 (orange), Site 204 (hot pink), and Site 220 (lime green) of the nucleocapsid protein. The frequencies of variants R203K, G204R, and A220V were high. (**D**) Site 85 (light pink) of the NSP2 protein. Many sequences showed a variant (T85I). The structures of spike, NSP12, the nucleocapsid protein, and NSP2 were collected from https://zhanglab.ccmb.med.umich.edu/COVID-19/. The protein structural figures were generated by the software PyMOL (http://www.pymol.org/, the accessed date: 16 January 2021).

We next explored the temporal and geographic dynamics of the variants with a frequency >15%, including: D614G (93.88%) and A222V (26.14%) in spike; A220V (25.96%), R203K (28.45%), and G204R (28.13%) in nucleocapsid; T85I (15.38%) in NSP2; Q57H (23.60%) in ORF3a; P323L (93.74%) in NSP12; V30L (26.01%) in ORF10 (Table 2). Figure 4B visualizes the temporal and geographical dynamics of 9 common variants on six continents during the COVID-19 pandemic, from December 2019 to January 2021. (i) Since the emergence of the spike variant D614G (Figure 5A) in January 2020 and the NSP12 variant P323L (Figure 5B) in January 2020, they have become the dominant variants across all continents, with an increasing prevalence over time (Figure 4B). (ii) During the pandemic, from July to December 2020, the prevalence of A222V in spike, A220V in nucleocapsid (Figure 5C), and V30L in ORF10 increased continuously in Europe and Africa, while their emergence in other continents was less common. (iii) Two neighboring variants in nucleocapsid, R203K and G204R (Figure 5C), showed roughly similar frequencies over time in the same geographic areas. (iv) Temporal trends of the NSP2 variant T85I (Figure 5D) and the ORF3a variant Q57H were distinct on different continents. From March to December 2020, the prevalence of T85I and Q57H was higher in North America than on other continents.

### 3.6. Global Surveillance of SARS-CoV-2 Clades

**Figure 6:**
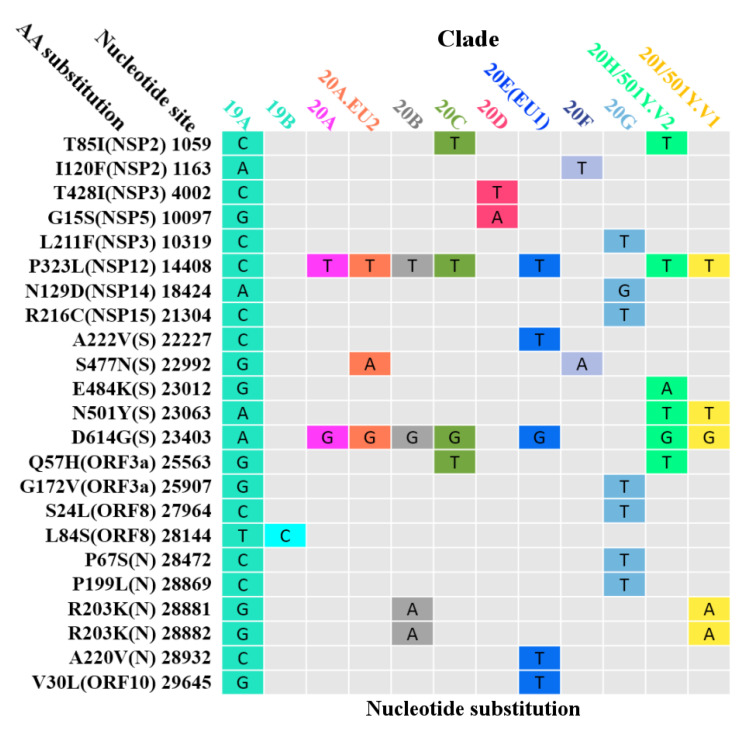
The substitution matrix at the nucleic acid level. Nucleotide substitutions showed in this figure are the main substitutions characterizing the SARS-CoV-2 clades.

The phylogenetic tree (Figure S2), performed based on the tree topology and variant clusters of 5488 complete SARS-CoV-2 nucleotide sequences, was divided into at least 12 different clades (19A, 19B, 20A, 20A.EU2, 20B, 20C, 20D, 20E (EU1), 20F, 20G, 20H/501Y.V2, and 20I/501Y.V1), as of January 2021. SARS-CoV-2 clades are classified based on nucleotide substitutions in the genome (Figure 6). For example, the 20A clade is featured with the variants D614G (spike) and P323L (NSP12), while the 20A.EU2 clade is defined by three variants of P323L (NSP12), S477N (spike), and D614G (spike). Furthermore, T85I (NSP2) and Q57H (ORF3a) were circulating with a high frequency in North America (Figure 4B). These two variants, plus P323L (NSP12) and D614G (spike), define the 20C clade. Moreover, the 20E (EU1) clade, with P323L (NSP12), A222V (spike), D614G (spike), A220V (nucleocapsid), and V30L (ORF10), gradually gained their predominance in Europe over time.

**Figure 7:**
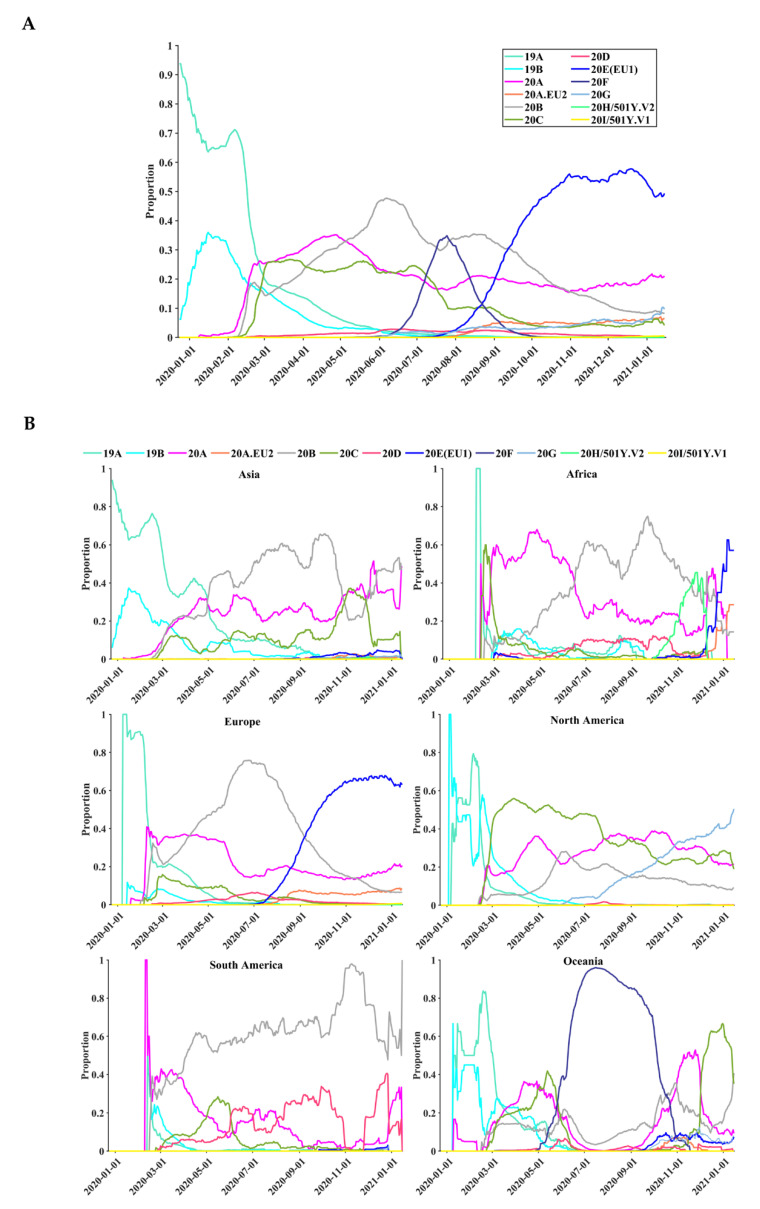
Temporal distributions of the 12 clades based on 260,673 complete SARS-CoV-2 nucleotide sequences. (**A**) The global distribution of the 12 clades over time. (**B**) The distribution of the 12 clades over time on six continents. The vertical axis represents the moving-window proportion, calculated by dividing the number of sequences belonging to a specific clade, 15 days before and after a specific date, by the total number of sequences in the period (31 days).

Temporal and geographical dynamics of 12 SARS-CoV-2 clades were observed on six continents based on 260,673 full-length genome sequences (Figure 7). Among the 12 different clades, the most prevalent clade was 20E (EU1) (*n* = 68,173, 26.15%), followed by 20B (*n* = 60,278, 23.12%), 20A (*n* = 56,801, 21.79%), 20C (*n* = 32,608, 12.51%), and others (*n* = 42,813, 16.43%) (Table S2). The 19A and 19B clades dominated the global infections from December 2019 to March 2020 (Figure 7A). Their dominant role in the global pandemic was subsequently replaced by the 20A clade. Since mid-September 2020, Clade 20E (EU1) became the dominant group worldwide. Furthermore, different continents exhibited different temporal dynamics (Figure 7B). For instance, the dominant clade in January 2021 was 20B in Asia, 20E (EU1) in Africa, 20E (EU1) in Europe, 20G in North America, 20B in South America, and 20C in Oceania. Clades 20H/501Y.V2 and 20I/501Y.V1, also known as B.1.351 and B.1.1.7, the two newly emerged clades, were mainly circulating in Africa and Europe, respectively.

## 4. Discussion

This study presents global surveillance of SARS-CoV-2 genetic diversity and circulating variants based on a large-scale dataset of 260,673 sequences sampled from December 2019 to January 2021. Based on this first one-year survey of genetic diversity, our study describes the temporal and geographical trends of circulating variants on different continents. Despite the proofreading activity of viral exoribonuclease, an increasing prevalence of key genetic variants, such as D614G in viral spike and P323L in NSP12, was observed in many countries and continents. Early surveillance of potentially evolving viral strains with higher transmission fitness and greater infectivity plays an important role in controlling the ongoing COVID-19 pandemic.

Among 26 viral proteins, the highest protein sequence diversity was observed in spike, nucleocapsid, ORF3a, and ORF10 (Table 1). As a critical target of many vaccines and neutralizing antibodies, the spike protein interacts with cellular receptors, such as angiotensin-converting enzyme 2 (ACE2), for viral entry [38,39]. Although whether the spike variants may weaken the performance of vaccines is still under evaluation [40,41], antibody therapeutics and vaccine development still need to consider the impact of spike variants on the antigenicity of the virus [42]. As an indispensable protein, nucleocapsid is the major target of primers and probes in many real-time reverse transcription polymerase chain reaction (RT-PCR) tests, while nucleotide substitutions in the primer binding region may affect the sensitivity of some diagnostic assays [43]. Although single nucleotide substitutions may not be an issue for solid assays that target multiple loci (*n* > 2) in the genome [44], close monitoring of SARS-CoV-2 nucleotide substitutions that are located within the primer and probe regions of RT-PCR assays remains important. ORF3a plays a role in virulence, ion channel formation, viral release, and apoptosis [45], while ORF10 is an accessory protein that is dispensable for viral replication in humans [46]. Our study presents that the highest substitution rate of the ORF10 protein appeared in Europe, followed by Africa (Figure 2B). Previous studies have reported the import of SARS-CoV-2 from Europe to Africa [47,48], which explains some similar patterns between Europe and Africa. To support surveillance, a paucity of SARS-CoV-2 sequence data from Africa requires further sequencing efforts on the African continent [47]. Additionally, other viral proteins are also known to interact with many host proteins that participate in multiple biological processes such as protein trafficking, translation, and transcription [49]. Genetic variants that promote viral fitness and escape may exert a potential impact on current diagnostic tests, vaccines, antiviral strategies, and immune responses [23,40,50]. Close monitoring of key viral variants is critical for the development and optimization of vaccines and antiviral therapies, according to previous experience from the management of viral infections such as influenza virus, respiratory syncytial virus, and human immunodeficiency virus [51,52,53,54,55,56,57].

Highly prevalent variants have been observed in SARS-CoV-2. A high prevalence of important variants, such as D614G (93.88%) in spike and P323L (93.74%) in NSP12, was confirmed by our large-scale study. Compared with wildtype D614, the D614G variant changes the dynamic structures of the viral spike to bind with human angiotensin-converting enzyme 2 [58] and increases transmission fitness with enhanced viral loads in the upper respiratory tract of SARS-CoV-2 cases [34]. The D614G variant is located at a region interfacing with the neighboring subunit, and it potentially alters the trimer stability of the viral spike to enhance membrane fusion and host entry [59]. In addition, D614G increases the replication fitness of SARS-CoV-2 in cell cultures and enhances viral transmission in hamsters [60]. Furthermore, D614G in spike is coevolving with P323L in NSP12, with strong allelic associations [16]. The coexistence of D614G (spike), P323L (NSP12), and C241U (5′-UTR) may contribute to increased transmission fitness [61]. The P323L variant might alter the secondary and tertiary structures of NSP12 to interact with NSP8, thereby affecting viral replication [62,63]. Additionally, other variants like S25L in NSP7, S194L, R203K, G204R, and T205I in nucleocapsid, T85I and I120F in NSP2, S477N in spike, and Q57H in ORF3a have also been confirmed by previous studies [62,64]. Although many SARS-CoV-2 variants have been observed, their associations with viral loads and infectivity need more investigation. Vaccines may need to be updated periodically to avoid the potential impact of newly arising SARS-CoV-2 variants on the clinical efficacy of the vaccines [65].

Key SARS-CoV-2 variants in certain clades may play an important role in viral transmission and adaptability. Monitoring the dynamics of SARS-CoV-2 clades is crucial because different viral clades, such as B.1.1.7, may be associated with viral loads and disease severity [66]. Due to the evolving nature of SARS-CoV-2, the emergence of some variants in B.1.1.7, B.1.351, and P.1 clades may affect viral transmissibility and antigenic profiles [65,67]. Based on large-scale full-length genome sequences, our findings showed distinct geographical and temporal patterns of SARS-CoV-2 clades on different continents (Figure 7). We observed the dynamic patterns of SARS-CoV-2 clades over time; for instance, Clade 20E (EU1) has become the dominant group worldwide since mid-September 2020. The 20H/501Y.V2 and 20I/501Y.V1 clades were observed in Africa in October 2020 and in Europe in November 2020, respectively. SARS-CoV-2 variants in different clades might exert an impact on some vaccines and antibodies [27,65,68]. Spike variants such as D614G and N501Y, contained in the 20H/501Y.V2 and 20I/501Y.V1 clades, may lead to changes in viral antigenicity that are harmful to monoclonal antibody therapies and vaccine protection [67], thereby mediating potential escape from vaccine response [41]. Since a cure against COVID-19 is still lacking, the existence of current and novel circulating clades that are globally overdispersed highlights the importance of rapid interventions to reduce viral transmissions [10].

There are limitations in our study. First, our analyses focused on genetic diversity over the past one-year period, while global surveillance of new clades or lineages is still needed during the ongoing COVID-19 pandemic. Second, patient information on disease severity, immune responses, and transmission history was largely lacking in our retrieved dataset. Future studies are needed to reveal potential associations of genetic variants with disease progression and transmission fitness. Third, a limited number of full-length genome sequences from Africa and Asia were deposited in the public databases, which limited our analyses to fully characterize the genetic diversity of SARS-CoV-2 in Africa and Asia. Future analyses should integrate more sequences from different countries and continents.

## 5. Conclusions

Despite its global spread, SARS-CoV-2 harbors only a small proportion of highly prevalent variants that determine the classification of viral clades or lineages. Based on a large-scale dataset of 260,673 genomic sequences, our study presents a comprehensive mapping of highly prevalent SARS-CoV-2 variants as well as the dynamic changes of clades during the one-year pandemic of COVID-19. Genomic surveillance is important for monitoring the genetic variants that may be resistant to current antiviral drugs and vaccines [35,69]. To effectively control the COVID-19 pandemic, further studies are still needed to evaluate the impact of highly prevalent variants on antiviral drugs and vaccines before their wide application.

## Figures and Tables

**Table 1:** Basic characteristics of SARS-CoV-2 proteins.

Protein	Gene	Protein Location	Protein Length	Polymorphic Sites	Substitution Rate (%)
NSP1	*ORF1a*	266–805	180	177	0.03
NSP2	*ORF1a*	806–2719	638	608	0.07
NSP3	*ORF1a*	2720–8554	1945	1752	0.03
NSP4	*ORF1a*	8555–10,054	500	419	0.02
3C-like protease (NSP5)	*ORF1a*	10,055–10,972	306	251	0.04
NSP6	*ORF1a*	10,973–11,842	290	256	0.06
NSP7	*ORF1a*	11,843–12,091	83	72	0.04
NSP8	*ORF1a*	12,092–12,685	198	176	0.03
NSP9	*ORF1a*	12,686–13,024	113	93	0.03
NSP10	*ORF1a*	13,025–13,441	139	105	0.01
NSP11	*ORF1a*	13,442–13,480	13	11	0.01
RNA-dependent RNA polymerase (NSP12)	*ORF1b*	13,442–13,468 13,468–16,236	932	715	0.13
Helicase (NSP13)	*ORF1b*	16,237–18,039	601	478	0.04
3′-to-5′ exonuclease (NSP14)	*ORF1b*	18,040–19,620	527	444	0.03
endoRNAse (NSP15)	*ORF1b*	19,621–20,658	346	313	0.04
2′-O-ribose methyltransferase (NSP16)	*ORF1b*	20,659–21,552	298	245	0.03
Spike glycoprotein (S)	*ORF2*	21,563–25,384	1273	1096	0.14
ORF3a	*ORF3a*	25,393–26,220	275	273	0.20
Envelope protein (E)	*ORF4*	26,245–26,472	75	72	0.02
Membrane protein (M)	*ORF5*	26,523–27,191	222	168	0.02
ORF6	*ORF6*	27,202–27,387	61	60	0.03
ORF7a	*ORF7a*	27,394–27,759	121	121	0.04
ORF7b	*ORF7b*	27,756–27,887	43	43	0.07
ORF8	*ORF8*	27,894–28,259	121	121	0.12
Nucleocapsid protein (N)	*ORF9*	28,274–29,533	419	378	0.31
ORF10	*ORF10*	29,558–29,674	38	37	0.72

**Table 2:** The overall prevalence of SARS-CoV-2 variants in a large-scale dataset of 260,673 sequences.

Genetic Variant	Variant Frequency (%)						
	Asia	Africa	Europe	North America	South America	Oceania	Total
T85I(NSP2)	10.23	5.72	3.26	52.63	8.84	5.96	15.38
I120F(NSP2)	3.43	0.00	2.12	0.02	0.00	72.98	5.23
M324I(NSP4)	0.25	0.41	4.38	0.08	0.09	0.11	2.84
L89F(NSP5)	0.23	0.03	0.08	15.73	0.06	0.33	3.75
L37F(NSP6)	19.93	4.93	6.84	3.28	3.09	4.78	6.52
A185S(NSP12)	0.15	0.34	4.35	0.04	0.00	0.12	2.81
P323L(NSP12)	71.54	91.83	96.04	92.87	97.29	91.22	93.74
V776L(NSP12)	0.18	0.34	4.33	0.24	0.00	0.12	2.84
K218R(NSP13)	0.15	0.34	4.32	0.02	0.00	0.11	2.78
E261D(NSP13)	0.20	0.58	4.39	0.08	0.03	0.11	2.84
H290Y(NSP13)	0.55	0.17	3.80	0.25	0.03	0.22	2.53
N129D(NSP14)	0.17	0.00	0.05	12.61	0.03	0.32	3.00
R216C(NSP16)	0.20	0.00	0.07	12.40	0.03	0.33	2.96
L18F(S)	0.32	0.28	18.78	0.30	0.34	0.12	12.08
A222V(S)	0.50	0.79	40.78	0.17	0.34	0.53	26.14
N439K(S)	0.45	0.03	3.90	0.01	0.06	0.06	2.52
S477N(S)	0.19	0.66	4.82	0.14	0.12	71.22	6.62
D614G(S)	71.91	93.91	96.11	93.10	97.26	91.26	93.88
Q57H(ORF3a)	24.97	12.34	11.86	59.86	14.94	8.08	23.59
G172V(ORF3a)	0.20	0.07	0.07	12.07	0.06	0.31	2.89
S24L(ORF8)	0.23	0.17	0.15	21.78	0.03	0.90	5.24
P67S(N)	0.17	0.03	0.11	12.21	0.06	0.33	2.94
S194L(N)	9.64	6.54	4.27	12.50	0.19	1.19	6.29
P199L(N)	0.29	0.21	2.29	12.49	0.06	0.40	4.42
R203K(N)	36.96	52.15	28.16	13.35	69.12	78.12	28.45
G204R(N)	36.73	51.62	27.71	13.31	69.07	78.08	28.13
A220V(N)	0.45	1.10	40.53	0.13	0.09	0.35	25.96
M234I(N)	0.50	0.55	4.77	0.61	3.64	0.30	3.28
A376T(N)	0.15	0.35	4.33	0.02	0.00	0.11	2.78
V30L(ORF10)	0.58	0.69	40.64	0.08	0.13	0.35	26.01

## Data Availability

SARS-CoV-2 whole-genome sequences were obtained from the Global Initiative on Sharing All Influenza Data (GISAID) (https://www.gisaid.org/, the accessed date: 16 January 2021).

## Supplementary Materials

The following are available online at https://www.mdpi.com/article/10.3390/biomedicines9040412/s1. A list of SARS-CoV-2 genome sequence acknowledgments is provided in Data S1. Table S1: Selective pressure analysis, Table S2: Distribution of clades, Figure S1: dN/dS ratios, Figure S2: Phylogenetic tree are provided as supplementary materials.
