# Supplementary material for: Genetic Diversity of SARS-CoV-2 over a One-Year Period of the COVID-19 Pandemic: A Global Perspective
Source: Biomedicines. 2021 Apr 11;9(4):412. doi: 10.3390/biomedicines9040412 (PMC8069977; doi:10.3390/biomedicines9040412)
Supplement: Supplementary file 1 [file biomedicines-09-00412-s001.zip › biomedicines-1156310-supplementary/biomedicines-1156310-supplementary-figures.docx]

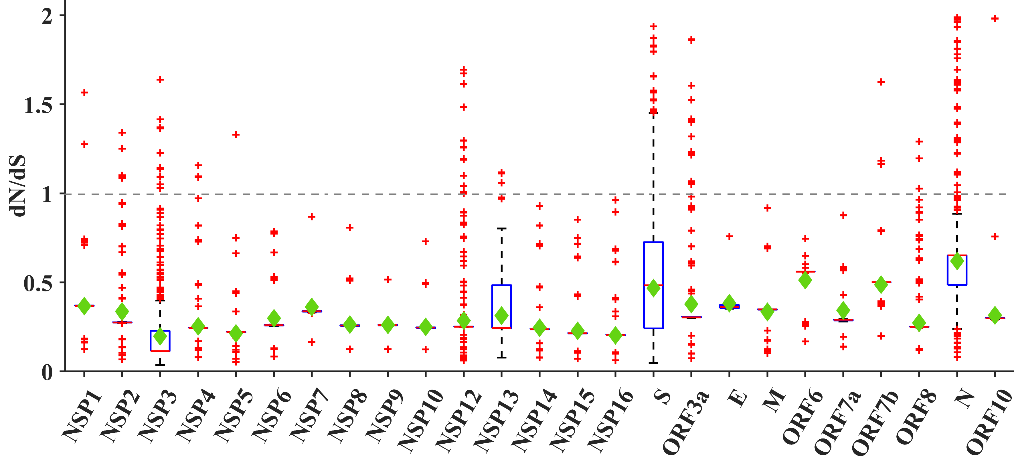


**Figure S1.** dN/dS ratios for the SARS-CoV-2 protein-coding genes. The protein-coding sequence with a dN/dS ratio >1 is defined as under positive selection. A dotted line was drawn on the figure to indicate the dN/dS ratio equal to 1. For each gene, the box spans the first and third quartiles. The green diamonds, the red horizontal lines, and the red plus signs represented the average values, the median values, and the outliers, respectively.


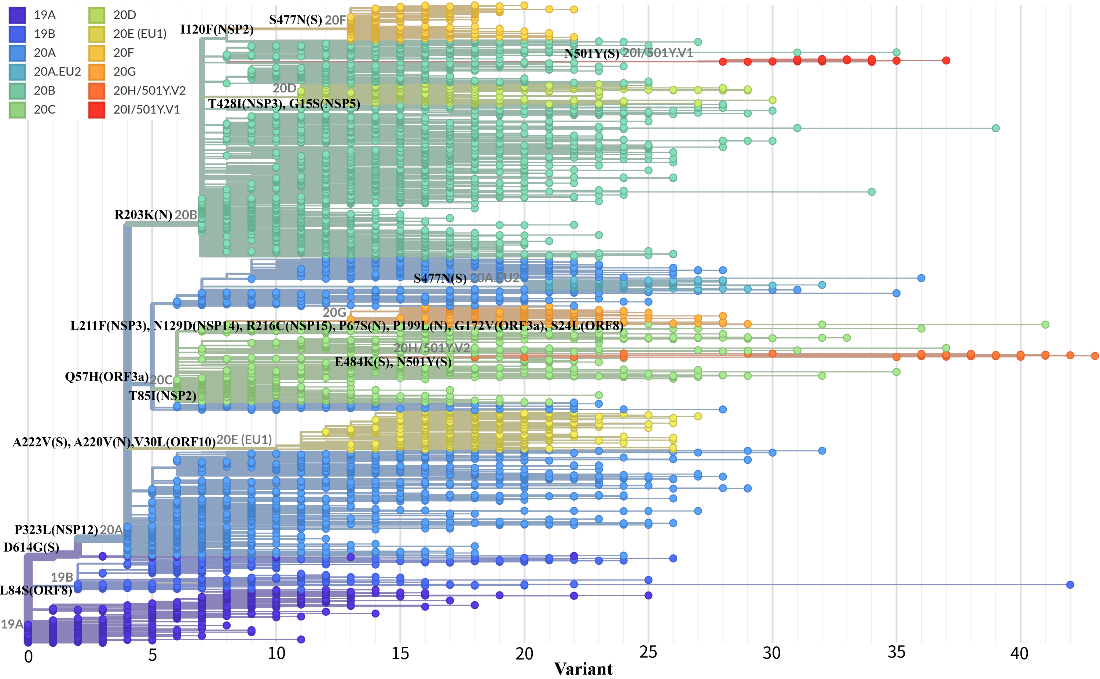


**Figure S2.** Phylogenetic tree of the SARS-CoV-2 full-genome sequences. The tree was constructed based on 5488 SARS-CoV-2 genome sequences using NextStrain tools (https://nextstrain.org, the accessed date: 2021/01/16).
