# Supplementary material for: Genetic Diversity of SARS-CoV-2 over a One-Year Period of the COVID-19 Pandemic: A Global Perspective
Source: Biomedicines. 2021 Apr 11;9(4):412. doi: 10.3390/biomedicines9040412 (PMC8069977; doi:10.3390/biomedicines9040412)
Supplement: Supplementary file 1 [file biomedicines-09-00412-s001.zip › biomedicines-1156310-supplementary/biomedicines-1156310-supplementary-tables.docx]

**Table S1.** Selective pressure analysis for SARS-CoV-2 protein-coding genes^1^.

| **Gene** | **Average** | **Median (IQR)** | **Proportion of Sequences With dN/dS >1 (%)** |
| --- | --- | --- | --- |
| *NSP1* | 0.37 | 0.37 (0.37–0.37) | <0.01 |
| *NSP2* | 0.34 | 0.28 (0.27–0.28) | 0.01 |
| *NSP3* | 0.20 | 0.11 (0.11–0.23) | 0.04 |
| *NSP4* | 0.25 | 0.24 (0.24–0.24) | <0.01 |
| *NSP5* | 0.21 | 0.22 (0.22–0.22) | <0.01 |
| *NSP6* | 0.30 | 0.26 (0.26–0.26) | 0.00 |
| *NSP7* | 0.36 | 0.34 (0.34–0.34) | 0.00 |
| *NSP8* | 0.26 | 0.26 (0.26–0.26) | 0.00 |
| *NSP9* | 0.26 | 0.26 (0.26–0.26) | 0.00 |
| *NSP10* | 0.25 | 0.25 (0.24–0.25) | 0.00 |
| *NSP12* | 0.29 | 0.25 (0.25–0.25) | 0.04 |
| *NSP13* | 0.31 | 0.24 (0.24–0.48) | <0.01 |
| *NSP14* | 0.25 | 0.24 (0.24–0.24) | 0.00 |
| *NSP15* | 0.23 | 0.21 (0.21–0.22) | 0.00 |
| *NSP16* | 0.20 | 0.20 (0.20–0.20) | 0.00 |
| *S* | 0.47 | 0.48 (0.24–0.73) | 0.86 |
| *ORF3a* | 0.38 | 0.30 (0.30–0.31) | 0.02 |
| *E* | 0.38 | 0.36 (0.35–0.37) | 0.00 |
| *M* | 0.34 | 0.35 (0.35–0.35) | 0.00 |
| *ORF6* | 0.51 | 0.56 (0.56–0.56) | 0.00 |
| *ORF7a* | 0.34 | 0.29 (0.29–0.29) | 0.00 |
| *ORF7b* | 0.49 | 0.50 (0.50–0.50) | <0.01 |
| *ORF8* | 0.27 | 0.25 (0.25–0.25) | 0.02 |
| *N* | 0.62 | 0.65 (0.49–0.65) | 1.11 |
| *ORF10* | 0.32 | 0.30 (0.30–0.30) | <0.01 |

^1^ For all viral protein-coding genes, the average and median, together with interquartile range (IQR) values of dN/dS, are listed.

**Table S2.** The number of genomic sequences in the major SARS-CoV-2 clades (*n* = 260,673).

| **Clade** | **Number of Sequences** | | | | | | |
| --- | --- | --- | --- | --- | --- | --- | --- |
|  | **Asia** | **Africa** | **Europe** | **North America** | **South America** | **Oceania** | **Total** |
| 19A | 2879 | 125 | 5248 | 1226 | 36 | 553 | 10,067 |
| 19B | 971 | 107 | 1288 | 2981 | 52 | 598 | 5997 |
| 20A | 3269 | 978 | 33,935 | 17,097 | 620 | 902 | 56,801 |
| 20A.EU2 | 23 | 10 | 7237 | 21 | 0 | 14 | 7305 |
| 20B | 4865 | 1293 | 43,102 | 7980 | 1894 | 1144 | 60,278 |
| 20C | 1345 | 80 | 5132 | 25,021 | 297 | 733 | 32,608 |
| 20D | 21 | 197 | 2482 | 133 | 372 | 92 | 3297 |
| 20E (EU1) | 58 | 16 | 68,033 | 21 | 3 | 42 | 68,173 |
| 20F | 0 | 0 | 0 | 0 | 0 | 8937 | 8937 |
| 20G | 17 | 0 | 75 | 6893 | 1 | 42 | 7028 |
| 20H/501Y.V2 | 2 | 83 | 8 | 0 | 0 | 0 | 93 |
| 20I/501Y.V1 | 0 | 0 | 89 | 0 | 0 | 0 | 89 |
